# Impuestos selectivos al consumo de bebidas azucaradas en América Latina y el Caribe^*^

**DOI:** 10.26633/RPSP.2021.124

**Published:** 2021-09-16

**Authors:** Rosa Carolina Sandoval, Maxime Roche, Itziar Belausteguigoitia, Miriam Alvarado, Luis Galicia, Fabio S. Gomes, Guillermo Paraje

**Affiliations:** 1 Organización Panamericana de la Salud Washington, D.C. Estados Unidos de América Organización Panamericana de la Salud, Washington, D.C. Estados Unidos de América.; 2 Universidad de Lancaster Lancaster Reino Unido Universidad de Lancaster, Lancaster, Reino Unido.; 3 Universidad de Cambridge Cambridge Reino Unido Universidad de Cambridge, Cambridge, Reino Unido.; 4 Ministerio de Salud Pública del Uruguay Montevideo Uruguay Ministerio de Salud Pública del Uruguay, Montevideo, Uruguay.; 5 Universidad Adolfo Ibáñez Santiago Chile Universidad Adolfo Ibáñez, Santiago, Chile.

**Keywords:** Enfermedades no transmisibles, política nutricional, economía de la salud, obesidad, legislación como asunto, Noncommunicable diseases, nutrition policy, health economics, obesity, legislation as topic, Doenças não transmissíveis, política nutricional, economia da saúde, obesidade, legislação como assunto

## Abstract

**Objetivo.:**

Caracterizar el diseño de los impuestos selectivos al consumo de bebidas azucaradas en América Latina y el Caribe, y evaluar las oportunidades de aumentar su impacto en el consumo y la salud.

**Métodos.:**

Se llevó a cabo una búsqueda y una evaluación exhaustivas de legislaciones vigentes a marzo del 2019, recopiladas mediante las herramientas de seguimiento ya existentes de la Organización Panamericana de la Salud y de la Organización Mundial de la Salud, fuentes secundarias, así como mediante una encuesta a ministerios de finanzas. El análisis se centró en el tipo de productos gravados y la estructura y la base de estos impuestos selectivos.

**Resultados.:**

De los 33 países evaluados, en 21 se aplican impuestos selectivos al consumo de bebidas azucaradas. En siete países también se aplican impuestos selectivos sobre el agua embotellada y en al menos cuatro, se aplican tales impuestos sobre las bebidas lácteas azucaradas. Diez de estos impuestos selectivos al consumo son de tipo *ad valorem* con algunas bases imponibles fijadas en las primeras etapas de la cadena de valor, siete son de tipo específico y cuatro son de estructura combinada o mixta. En tres países se aplican impuestos selectivos al consumo en función de la concentración de azúcares del producto.

**Conclusiones.:**

Si bien el número de países en que se aplican impuestos selectivos al consumo de bebidas azucaradas es prometedor, existe una gran heterogeneidad en su diseño en cuanto a la estructura, la base imponible y los productos gravados. Se podrían aprovechar aún más los impuestos selectivos existentes para tener un mayor impacto sobre el consumo de bebidas azucaradas y la salud si se incluyeran todas las categorías de bebidas azucaradas, excluyendo el agua embotellada, y si se recurriera más a impuestos específicos ajustados regularmente según la inflación y basados posiblemente en la concentración de azúcares del producto. Todos los países se beneficiarían si hubiera mayor orientación. Futuras investigaciones deberían tener como objetivo abordar esta brecha.

## Abstract (en)

**Objective.:**

To characterize the design of excise taxes on sugar-sweetened beverages (SSBs) in Latin America and the Caribbean and assess opportunities to increase their impact on SSB consumption and health.

**Methods.:**

A comprehensive search and review of the legislation in effect as of March 2019, collected through existing Pan American Health Organization and World Health Organization monitoring tools, secondary sources, and surveying ministries of finance. The analysis focused on the type of products taxed, and the structure and base of these excise taxes.

**Results.:**

Out of the 33 countries analyzed, 21 apply excise taxes on SSBs. Seven countries also apply excise taxes on bottled water and at least four include sugar-sweetened milk drinks. Ten of these excise taxes are ad valorem with some tax bases set early in the value chain, seven are amount-specific, and four have either a combined or mixed structure. Three countries apply excise taxes based on sugar concentration.

**Conclusions.:**

While the number of countries applying excise taxes on SSBs is promising, there is great heterogeneity in design in terms of structure, tax base, and products taxed. Existing excise taxes could be further leveraged to improve their impact on SSB consumption and health by including all categories of SSBs, excluding bottled water, and relying more on amount-specific taxes regularly adjusted for inflation and possibly based on sugar concentration. All countries would benefit from additional guidance. Future research should aim to address this gap.

## RESUMO

**Objetivo.:**

Caracterizar o modelo dos impostos especiais de consumo sobre bebidas açucaradas na América Latina e no Caribe e avaliar oportunidades para aumentar o impacto desses impostos no consumo de bebidas açucaradas e na saúde.

**Métodos.:**

Realizou-se uma pesquisa ampla e a análise de legislações vigentes em março de 2019, com informações obtidas por meio de instrumentos de monitoramento da Organização Pan-Americana da Saúde (OPAS) e da Organização Mundial da Saúde (OMS) já existentes, fontes secundárias e levantamento junto aos ministérios da Fazenda. A análise centrou-se no tipo de produtos tributados e na estrutura e base desses impostos especiais de consumo.

**Resultados.:**

Dos 33 países analisados, 21 aplicam impostos especiais de consumo sobre bebidas açucaradas. Em sete países os impostos especiais de consumo incidem também sobre água engarrafada e, em pelo menos quatro, incluem bebidas lácteas açucaradas. Dez desses tributos especiais são ad valorem com algumas bases tributárias estabelecidas no início da cadeia de valor, sete são de tipo específico e quatro têm uma estrutura combinada ou mista. Em três países os impostos especiais são estabelecidos com base na concentração de açúcares do produto.

**Conclusões.:**

Apesar do número promissor de países com impostos especiais de consumo sobre bebidas açucaradas, verifica-se grande heterogeneidade nos modelos de tributação em termos de estrutura, base tributária e produtos tributados. Os impostos especiais de consumo vigentes poderiam ser mais bem aproveitados para aumentar o impacto no consumo de bebidas açucaradas e na saúde: incluir todas as categorias de bebidas açucaradas, excluir água engarrafada e recorrer mais a impostos de tipo específico com a correção periódica pela inflação e, possivelmente, com base na concentração de açúcares do produto. Todos os países se beneficiariam em receber mais orientação. Pesquisas futuras devem ter como objetivo abordar essa lacuna.

En toda América Latina y el Caribe, como en casi todo el mundo, la carga de enfermedades no transmisibles (ENT) es elevada y sigue en aumento. Al año 2016, tres de cada cuatro muertes en esta región fueron provocadas por ENT, y de ellas, 43% se registraron en personas de menos de 70 años (1). Las cuatro ENT principales (cáncer, diabetes mellitus, enfermedades cardiovasculares y enfermedades respiratorias crónicas) representaron 2,15 millones de muertes, es decir, tres cuartas partes de todas las muertes provocadas por ENT (1). Se ha estimado que, a escala mundial, el costo de esas cuatro ENT ascendió a US$ 3,8 billones en el 2010, y las proyecciones lo sitúan en US$ 7 billones para el 2030 (2).

La Organización Mundial de la Salud (OMS) ha establecido una serie de soluciones costo-eficaces y basadas en la evidencia para prevenir y controlar las ENT (3, 4). Entre ellas cabe mencionar la recomendación de reducir el consumo de azúcares mediante la tributación eficaz de las bebidas azucaradas (3–5). Estas se definen como todos los tipos de bebidas sin alcohol que contienen azúcares libres, entre las que se incluyen los refrescos gaseosos o no gaseosos, los jugos y bebidas de frutas y verduras, los concentrados líquidos y en polvo, el agua saborizada, las bebidas energizantes y deportivas, el té listo para beber, el café listo para beber y las bebidas lácteas saborizadas. Estas bebidas proporcionan un valor nutritivo limitado, pueden conducir a una ingesta calórica excesiva y se las ha relacionado con resultados de salud negativos (6). Es más, se las ha señalado como el principal factor de la epidemia de obesidad (7) y en exámenes sistemáticos se ha demostrado que su consumo se vincula con el aumento de peso en niños y adultos (8), un riesgo mayor de hipertensión y cardiopatías coronarias (9), y una mayor incidencia de diabetes de tipo 2 (10).

América Latina y el Caribe tiene la mayor mortalidad absoluta del mundo relacionada con el consumo de bebidas azucaradas, con unas 159 muertes por millón de adultos (en comparación con 48 muertes por millón de adultos a nivel mundial), de las que 80% se vinculan con la diabetes (11). El consumo diario medio de bebidas azucaradas por adulto en América Latina y el Caribe es el más alto del mundo, en particular en el Caribe y Centroamérica (en promedio 1,93 y 1,61 raciones diarias de ocho onzas por adulto, respectivamente, en comparación con 0,58 a nivel mundial) (12). Además, los niveles de obesidad son mayores que en cualquier otra parte del mundo (13). La aplicación de impuestos selectivos sobre las bebidas azucaradas es una opción de política prometedora para reducir el consumo de dichas bebidas y la carga de las ENT. Varias evaluaciones de los impuestos selectivos sobre las bebidas azucaradas aplicados en América Latina y el Caribe han demostrado que se vinculan con reducciones en las ventas y el consumo de dichas bebidas (14–17). Por ejemplo, se ha demostrado que el impuesto selectivo de 1 peso por litro introducido en México en el 2014 redujo las compras de bebidas azucaradas en un promedio anual de 7,6% durante los dos primeros años posteriores a su establecimiento (14). El impuesto selectivo *ad valorem* en dos niveles adoptado en Chile se ha vinculado con una reducción de 3,4% en el volumen mensual promedio adquirido por hogar en cuanto a las bebidas azucaradas con la mayor concentración de azúcares (15). El impuesto selectivo *ad valorem* en Barbados se ha vinculado con una reducción de 4,3% en las ventas de bebidas azucaradas durante su primer año (16). Por último, en un estudio de simulación se constató que tan solo en México se prevé que el actual impuesto selectivo sobre las bebidas azucaradas prevendrá entre 86.000 y 134.000 casos de diabetes de tipo 2 para el 2030 y reducirá la prevalencia de la obesidad en 2,5%, lo cual destaca el potencial que tienen los impuestos selectivos sobre las bebidas azucaradas de contribuir a los esfuerzos para prevenir las ENT (17).

Centramos nuestro análisis particularmente en los impuestos selectivos, pues tienen el mayor potencial, desde la óptica de la salud, en comparación con otros tipos de gravámenes indirectos (por ejemplo, impuestos sobre las ventas aplicados en el punto de compra [el cajero], o sobre el valor agregado). En efecto, los impuestos selectivos permiten a los responsables de las políticas enfocarse en determinados productos y aumentar su precio, con lo cual los vuelven relativamente menos asequibles que otros bienes y servicios (18). Un impuesto selectivo se aplica sobre un bien determinado, importado o de producción local, y puede ser de monto específico (basado en el volumen de la bebida o su contenido de azúcar; por ejemplo, US$ 0,10 por litro) o *ad valorem* (basado en un porcentaje del valor de la bebida; por ejemplo, 10% del precio de fábrica) (19). Se ha dicho que los impuestos selectivos sobre las bebidas azucaradas ofrecen un beneficio triple para los gobiernos, porque 1) mejoran la salud de la población, 2) generan ingresos tributarios y 3) pueden reducir los correspondientes costos de atención de salud y pérdidas de productividad a largo plazo (20, 21).

Si bien es posible que haya enseñanzas de la tributación del tabaco que también puedan aplicarse en el caso de las bebidas azucaradas, la orientación en cuanto a las mejores prácticas relativas a la tributación de dichas bebidas todavía se está elaborando. La mayoría de las evaluaciones de los impuestos selectivos sobre las bebidas azucaradas se centran únicamente en un país a la vez y se concentran en los impuestos recientemente implementados, aplicados con fundamentos de salud explícitos. Al momento de realizarse el presente análisis no estaba claro cuántos países aplican impuestos selectivos sobre las bebidas azucaradas en toda América Latina y el Caribe, o de qué manera estos varían en su diseño. Es importante caracterizar de manera integral el entorno de los actuales impuestos selectivos sobre las bebidas azucaradas a nivel de país, debido a lo siguiente: 1) el alcance de la epidemia mundial de las ENT, 2) el reconocimiento de los impuestos selectivos sobre las bebidas azucaradas como intervención costo-eficaz y basada en la evidencia, y 3) el mayor interés de los responsables de las políticas en distintos lugares del mundo en modificar o introducir dichos impuestos.

Los objetivos de este análisis son ofrecer la primera revisión exhaustiva de alcance regional de los impuestos selectivos sobre las bebidas azucaradas en América Latina y el Caribe, caracterizar la actual aplicación de dichos impuestos y evaluar las oportunidades de mejorar su impacto sobre el consumo de bebidas azucaradas y la salud.

## MATERIALES Y MÉTODOS

Efectuamos una búsqueda integral de la legislación (decretos, leyes, leyes sobre impuestos selectivos y edictos y decretos-ley) sobre los impuestos selectivos aplicados a las bebidas no alcohólicas en los 33 Estados Miembros de América Latina y el Caribe de la Organización Panamericana de la Salud (OPS), Oficina Regional de la OMS para la Región de las Américas. Examinamos la legislación ya recopilada a través de las herramientas de seguimiento existentes de la OPS/OMS —el examen de las políticas de nutrición a escala mundial de la OMS, el informe de la OMS sobre la epidemia mundial de tabaquismo, el Sistema Mundial de Información sobre el Alcohol y la Salud de la OMS y la encuesta de capacidad de país de las ENT de la OPS— y realizamos búsquedas en los sitios web de distintos parlamentos y ministerios de economía y también en bases de datos jurídicas. Por último, mediante una encuesta realizada entre marzo y diciembre del 2019 (en adelante, encuesta de la OPS sobre los impuestos a las bebidas azucaradas) se solicitó información sobre los impuestos aplicados a las bebidas no alcohólicas directamente a los profesionales designados oficialmente de los ministerios de economía. La encuesta fue completada por 27 Estados Miembros de la OPS de América Latina y el Caribe (todos excepto Argentina, Bahamas, el Estado Plurinacional de Bolivia, Costa Rica, Haití y Nicaragua).

### Criterios de inclusión

Buscamos impuestos selectivos aplicados a las bebidas sin alcohol para identificar y analizar los aplicados a las bebidas azucaradas, ya fuera que excluyeran o no a las bebidas no azucaradas como el agua embotellada y las bebidas endulzadas artificialmente. Incluimos legislación en la que se indica la estructura de tasas de los impuestos selectivos, así como leyes en las que se describe la base imponible sobre la cual se aplican las tasas de dichos impuestos. Los datos presentados se basan en legislación vigente al 31 de marzo del 2019.

### Extracción de datos

Extrajimos la siguiente información de la legislación de cada país: nombre, año de la actualización más reciente, estructura tributaria (*ad valorem*, monto específico, mixta o combinada) y base imponible. En el caso de los impuestos *ad valorem*, evaluamos si la base imponible se define en los primeros tramos de la cadena de valor (por ejemplo, precio de fábrica) o en tramos subsiguientes (por ejemplo, precio minorista). En relación con los impuestos de monto específico, evaluamos si se ajustan automáticamente en función de la inflación o de otros indicadores económicos.

Además, elaboramos un grupo sencillo de indicadores para reflejar la heterogeneidad de diseño de los impuestos selectivos en los distintos países. Evaluamos si el diseño es uniforme o escalonado, si el contenido de azúcar se usa como base imponible, y si el impuesto se aplica sobre el agua embotellada, para captar cualquier diferenciación entre las bebidas azucaradas y las no azucaradas. Por último, evaluamos si la definición de productos imponibles incluye las bebidas lácteas azucaradas, las energizantes y los polvos, concentrados o jarabes usados para hacer bebidas azucaradas mediante el agregado de agua con o sin gas, a fin de evaluar si cada impuesto se aplica a una amplia gama de bebidas azucaradas o si incluye lagunas fiscales que puedan promover sustituciones no deseadas y elusión fiscal.

## RESULTADOS

Constatamos que al mes de marzo del 2019, 21 Estados Miembros de la OPS de América Latina y el Caribe aplicaban impuestos selectivos sobre las bebidas azucaradas, con una proporción menor en el Caribe (6/13, información no disponible en el caso de Haití) que en América Latina (15/19). Once países (Colombia, Cuba, la República Dominicana, la República Bolivariana de Venezuela y la mayoría de los países del Caribe) no aplicaban un impuesto selectivo sobre dichas bebidas (cuadro 1).

### Productos imponibles

Los países que aplican impuestos selectivos sobre las bebidas azucaradas definen los productos imponibles de diferentes maneras. Todos los países del Caribe y algunos de los latinoamericanos (en su mayoría en Centroamérica) usan códigos arancelarios armonizados —nomenclatura internacional estandarizada para clasificar los productos objeto de comercio— para definir los productos imponibles, si bien el rango de códigos arancelarios incluidos es amplio. Es importante destacar que si bien algunos de esos códigos se definen en función de los azúcares añadidos (por ejemplo, 22.02: “Agua, incluidas el agua mineral y la gaseada, con adición de azúcar u otro edulcorante”), otros no (23), y es posible que los países deban elaborar códigos específicos más detallados para diferenciar entre bebidas azucaradas. La mayoría de los demás países latinoamericanos han elaborado definiciones de los productos imponibles sobre la base del tipo de bebida.

De todos los países que aplican impuestos selectivos sobre las bebidas azucaradas, siete los aplican también al agua embotellada. Al menos 14 incluyen uno o varios de los siguientes productos en su lista de productos imponibles: polvos, concentrados o jarabes usados para hacer bebidas azucaradas agregando agua gaseosa o no gaseosa. Todos los países que establecen impuestos selectivos sobre las bebidas azucaradas los aplican sobre las bebidas energizantes. Por último, por lo menos cuatro (Barbados, Panamá, Perú y San Vicente y las Granadinas) aplican impuestos selectivos sobre las bebidas lácteas azucaradas (cuadro 2).

### Estructura tributaria

Los actuales impuestos selectivos sobre las bebidas azucaradas se basan en una variada gama de estructuras tributarias. Diez países usan exclusivamente impuestos *ad valorem* y siete usan impuestos de monto específico. Dominica y Ecuador utilizan una estructura de impuesto selectivo combinada. Dominica aplica un impuesto *ad valorem* sobre las bebidas azucaradas excepto las gaseosas, que están sujetas a un impuesto de monto específico. Ecuador aplica uno de monto específico sobre las bebidas azucaradas con una concentración de azúcar por encima de un umbral especificado y otro *ad valorem* sobre las bebidas azucaradas situadas por debajo de aquel umbral. Todas las bebidas energizantes (independientemente de su concentración de azúcar) están gravadas con el impuesto *ad valorem*. Por último, El Salvador y México usan una estructura de impuesto selectivo mixta sobre las bebidas energizantes. México utiliza principalmente uno de monto específico y aplica otro *ad valorem* adicional a las bebidas energizantes, mientras que El Salvador utiliza principalmente un impuesto *ad valorem* y aplica otro adicional de monto específico a las bebidas energizantes (cuadro 1).

En cinco países (Estado Plurinacional de Bolivia, Costa Rica, Ecuador, Honduras y México) de los once que incluyen un componente de monto específico, la legislación estipula un ajuste automático periódico del impuesto selectivo correspondiente (cuadro 2).

Once de los impuestos selectivos sobre las bebidas azucaradas establecidos en América Latina y el Caribe aplican tasas tributarias múltiples (diseño escalonado), en lugar de una sola tasa para todas las bebidas azucaradas sujetas al impuesto selectivo (diseño uniforme). Las tasas escalonadas se definen con mayor frecuencia por tipo de bebida o código arancelario armonizado. Otros ejemplos incluyen diseños escalonados definidos por umbrales de concentración de azúcar (por ejemplo, Perú y Chile), una combinación de tipo de bebida y contenido de azúcar (por ejemplo, Ecuador) o una combinación de tipo de bebida y concentración de jugo de fruta (por ejemplo, Argentina). Diez países aplican impuestos selectivos uniformes (cuadro 2).

**CUADRO 1.** Resumen de los impuestos selectivos sobre las bebidas azucaradas en América Latina y el Caribe (sobre la base de la legislación vigente al 31 de marzo del 2019)

País	Aplica impuestos selectivos sobre las bebidas azucaradas	Estructura tributaria	Año de la última actualización de la legislación	Legislación
**América Latina**				
Argentina	Sí	*Ad valorem*	2018	Ley de Impuestos Internos N 24.674 Decreto N 2682-1979
Bolivia (Estado Plurinacional de)	Sí	Monto específico	2018	Directorio Actualización de la Alícuotas Específicas del ICE para la gestión 2019 N 101800000031 Decreto Supremo 0744 de diciembre 2010
Brasil	Sí	*Ad valorem*	2016	Decreto N 8.442 de abril 2015 Decreto N 8.950 de diciembre 2016
Chile	Sí	*Ad valorem*	2017	Ley N 21.045, D.O. 3 nov 2017 Decreto de ley N 825
Colombia	No			
Costa Rica	Sí	Monto específico	2018	Ley de Simplificación y Eficiencia Tributarias N 8114 Decreto Ejecutivo N 41495 de diciembre 2018
Cuba	No			
Ecuador	Sí	Combinado^b^	2016	Ley Orgánica para el Equilibrio de las Finanzas Públicas, 2016
El Salvador	Sí	*Ad valorem* (mixto sobre bebidas energizantes)^c^	2010	Decreto N 237 del 2010
Guatemala	Sí	Monto específico	2002	Decreto N 09-2002; Expediente acumulado 404 y 492-2002
Honduras	Sí	Monto específico	2019	Acuerdo Número 163-2019
México	Sí	Monto específico (mixto sobre bebidas energizantes)^c^	2018	Ley del impuesto Especial sobre Producción y Servicios, DOF 28-12-2018
Nicaragua	Sí	*Ad valorem*	2019	Ley de Concertación Tributaria N 987
Panamá	Sí	*Ad valorem*	1995	Ley N 45, 14 nov 1995
Paraguay	Sí	*Ad valorem*	2015	Ley N 5538/15; Ley N 125/91
Perú	Sí	*Ad valorem*	2018	Decreto Supremo N 091-2018-EF Título II - Decreto Supremo N 055-99-EF
Rep. Dominicana	No			
Uruguay	Sí	Monto específico^d^	2019	Decreto 20.019; Decreto N 96/990; IMESI título 11-1996
Venezuela (República Bolivariana de)	No			
**Caribe**				
Antigua y Barbuda	No			
Bahamas	No			
Barbados	Sí	*Ad valorem*	2017	Excise Tax (Amendment) (NO.3) Regulations 2017 Excise Tax Act 2015-32
Belice	Sí	Monto específico	2017	Customs and Excise Duties (Amendment) Act N 29, 2017 Customs and Excise Duties (Amendment) Act N 8, 2016
Dominica	Sí	Combinado^b^	2015	Excise Tax (Amendment), SRO N 28 of 2015 Excise Tax Act 8, 2005
Granada	No			
Guyana	No			
Haití^a^	…			
Jamaica	No			
Saint Kitts y Nevis	Sí	*Ad valorem*	2010	Excise Tax Act No. 4, 2010
San Vicente y las Granadinas	Sí	*Ad valorem*	2009	Excise Tax Act Chapter 430 SRO 2
Santa Lucía	No			
Suriname	Sí	Monto específico	2006	S.B. 2006 no. 27 wijz. Wet Accijns Alcoholvrije Dranken
Trinidad y Tabago	No			

… : información no disponible

a Haití: El país no participó en la encuesta de la OPS sobre los impuestos a las bebidas azucaradas en el 2019. En nuestra búsqueda de legislación encontramos una ley de 1971 sobre impuestos selectivos (“Loi sur le Droit d’Accise, du 21 octobre 1971”) que establece un impuesto selectivo de monto específico sobre las bebidas gaseosas tanto importadas como de producción local. Sin embargo, en un informe posterior de la Organización Mundial del Comercio se indica que a junio del 2015 dicho impuesto tenía una estructura diferente para esas bebidas según fueran importadas (de monto específico) o de producción local (*ad valorem*), lo cual podría constituir una violación del tratamiento nacional (22). No encontramos legislación ni información más reciente sobre ese impuesto. Ante su potencial naturaleza discriminatoria por diferenciar entre bebidas importadas o de producción local, y en vista de la falta de información, decidimos no incluirlo en nuestro análisis.

b Combinado: Por lo menos un tipo de bebida no alcohólica está gravado con un impuesto selectivo *ad valorem* y por lo menos otro tipo lo está con uno de monto específico. Ningún tipo de bebida está gravado con ambos.

c Mixto: Por lo menos un tipo de bebida no alcohólica está gravado tanto con un impuesto selectivo *ad valorem* como con uno de monto específico. En El Salvador y México únicamente las bebidas energizantes están gravadas por un sistema de impuesto selectivo mixto.

d Uruguay: El impuesto selectivo sobre las bebidas azucaradas está estructurado como impuesto *ad valorem* aplicado sobre una base imponible de montos fijos—“precios fictos”—por volumen, que varían por tipo de bebida, con lo cual funciona de hecho como un impuesto específico, y así se lo ha clasificado en el presente análisis.

***Fuente:*** Preparado por los autores sobre la base de los datos del estudio.

**CUADRO 2.** Información acerca del diseño de los impuestos selectivos sobre las bebidas azucaradas en América Latina y el Caribe (sobre la base de la legislación vigente al 31 de marzo del 2019)

País que aplica impuestos selectivos sobre las bebidas azucaradas	Los aplica al agua embotellada	Los aplica a los polvos, concentrados o jarabes^e^	Los aplica a las bebidas energi-zantes	Los aplica a las bebidas lácteas azucaradas (código arancelario armonizado: 040299)^e^	Base imponible *ad valorem* para bebidas de producción local	Ajuste automático del impuesto selectivo de monto especifico según la inflación u otros indicadores económicose	Impuesto selectivo basado en el contenido de azúcar	Tasa tributaria uniforme (No = escalonada)
**Impuesto selectivo con estructura *ad valorem***								
Argentina	Sí	Sí	Sí	…	Precio minorista excluido IVA	NA	No	No
Barbados	No	Sí	Sí	Sí	Precio de fábrica	NA	No	Sí
Brasil	No^d^	Sí	Sí	No	Precio de fábrica	NA	No	Sí
Chile	No	Sí	Sí	No	Precio minorista excluido IVA	NA	Sí^i^	No
Nicaragua	Sí	Sí	Sí	…	Precio minorista	NA	No	No^j^
Panamá	No	Sí	Sí	Sí	Precio minorista	NA	No	Sí
Paraguay	No	No	Sí	No	Precio de fábrica	NA	No	Sí
Perú	No	No	Sí	Sí	Precio minorista excluido IVA e imp. selectivo	NA	Sí^i^	No
Saint Kitts y Nevis	No	No	Sí	No	Precio minorista excluido IVA	NA	No	Sí
San Vicente y las Granadinas	No	No	Sí	Sí	Precio minorista excluido IVA	NA	No	Sí
**Impuesto selectivo con estructura de monto específico**								
Belice	Sí	No	Sí	No	NA	No	No	Sí
Bolivia (Estado Plurinacional de)	No	…	Sí	…	NA	Sí	No	No
Costa Rica	Sí	Sí	Sí	…	NA	Sí	No	No
Guatemala	Sí	Sí	Sí	No	NA	No	No	No
Honduras	No	No	Sí	No	NA	Sí	No	Sí
Suriname	Sí	Sí	Sí	No	NA	No	No	Sí
Uruguay^a^	Sí	Sí	Sí	No	Base imponible fija “precios fictos”	No^g^	No	No
**Impuesto selectivo con estructura combinada^b^**								
Dominica	No	Sí	Sí	No	Precio de fábrica	No	No	No
Ecuador	No	Sí	Sí	No	Precio minorista excluido IVA e imp. selectivo	Sí	Sí	No
**Impuesto selectivo con estructura mixta^c^**								
El Salvador	No	Sí	Sí	No	Precio minorista excluido IVA e imp. selectivo	No^h^	No	No
México	No	Sí	Sí	No	Precio de fábrica^f^	Sí	No	Sí

… : información no disponible

NA: no se aplica IVA: impuesto sobre el valor agregado

a Uruguay: El impuesto selectivo sobre las bebidas azucaradas está estructurado como impuesto *ad valorem* aplicado sobre una base imponible de montos fijos —“precios fictos”— por volumen, que varían por tipo de bebida, con lo cual funciona de hecho como un impuesto específico, y así se lo ha clasificado en el presente análisis.

b Combinado: Por lo menos un tipo de bebida no alcohólica está gravado con un impuesto selectivo *ad valorem* y por lo menos otro tipo lo está con uno de monto específico. Ningún tipo de bebida está gravado con ambos.

c Mixto: Por lo menos un tipo de bebida no alcohólica está gravado tanto con un impuesto selectivo *ad valorem* como con uno de monto específico. En El Salvador y México únicamente las bebidas energizantes están gravadas por un sistema de impuesto selectivo mixto.

d Brasil: Solamente las aguas minerales naturales están exentas de impuesto selectivo.

e Información disponible únicamente para los países que respondieron a la encuesta de la OPS sobre los impuestos a las bebidas azucaradas. En el caso de Argentina, el Estado Plurinacional de Bolivia, Costa Rica y Nicaragua, en algunos casos, se pudo extraer la información de la legislación.

f México: El componente *ad valorem* se aplica únicamente a las bebidas energizantes.

g Uruguay: Los montos de la base imponible fija —“precios fictos”— se suelen ajustar anualmente, pero eso no es un requisito dispuesto por ley.

h El Salvador: El componente de monto específico se aplica únicamente a las bebidas energizantes.

i Chile y Perú: Diseño escalonado con distintas tasas tributarias *ad valorem* definidas por umbrales de concentración de azúcar.

j Nicaragua: La tasa tributaria *ad valorem* es uniforme para las bebidas azucaradas, pero al agua mineral se le aplica una tasa menor.

***Fuente:*** Preparado por los autores sobre la base de los datos del estudio.

### Base imponible

Para los impuestos selectivos *ad valorem*, la base imponible se define como el valor del producto gravado, que se puede aplicar en distintos tramos de la cadena de valor. En los casos en que la base imponible para las bebidas de producción local se determina en fases iniciales de dicha cadena, como el precio de fábrica, según ocurre en cinco países (Barbados, Brasil, Dominica, México y Paraguay), las tasas del impuesto *ad valorem* se aplican a un valor menor, lo cual reduce el impacto del impuesto en los precios minoristas finales. Los otros nueve países que tienen un componente *ad valorem* usan una base imponible para las bebidas de producción local que se define en tramos posteriores de la cadena de valor, más cerca del precio minorista final (cuadro 2). En el caso de las bebidas importadas, los países que aplican impuestos selectivos *ad valorem* utilizan como base imponible el valor de costo, seguro y flete (CIF, por su sigla en inglés) y, cuando corresponde, los derechos de importación y aduaneros. El valor CIF se usa en la mayoría de los casos como base para los derechos de importación y se define como el valor de la consignación sin descargar, que comprende los costos del propio producto, del seguro y del transporte y descarga. Esa base imponible, establecida en tramos iniciales de la cadena de valor, reduce el impacto del impuesto en los precios minoristas finales.

En el caso de los impuestos selectivos de monto específico, la base imponible se puede definir según el volumen de la bebida o el contenido de azúcar. Casi todos los países con un componente de monto específico utilizan como base imponible el volumen de la bebida. Solamente Ecuador utiliza el contenido de azúcar como base imponible para su impuesto selectivo de monto específico de US$ 0,18 por 100 gramos de azúcares para bebidas de más de 25 gramos de azúcares por litro (excepto las energizantes). Además, Chile y Perú utilizan un diseño escalonado para su impuesto selectivo *ad valorem* con distintas tasas tributarias definidas por umbrales de concentración de azúcar (cuadro 2).

## DISCUSIÓN

La mayoría de los países de América Latina y el Caribe aplican impuestos selectivos sobre las bebidas azucaradas. Sin embargo, algunos de esos impuestos llevan más de un decenio sin actualizarse y muchos no están optimizados para alcanzar objetivos de salud.

### Consideraciones clave en el diseño de impuestos selectivos sobre las bebidas azucaradas

Partiendo de la base de las mejores prácticas sobre la tributación del tabaco, establecimos algunas consideraciones clave para las distintas estructuras de impuestos selectivos observadas. Se ha demostrado que los impuestos selectivos *ad valorem* sobre los productos tabacaleros amplían la brecha entre el precio de los productos menos y más caros, lo cual induce al consumidor a optar por marcas más baratas y menoscaba los potenciales beneficios de salud del impuesto (18). La evidencia emergente sugiere que los impuestos selectivos *ad valorem* sobre las bebidas azucaradas pueden tener un efecto similar (16). Además, los impuestos selectivos *ad valorem* aplicados en función de una base imponible establecida en tramos iniciales de la cadena de valor, como el precio de fábrica para las bebidas de producción local y el valor CIF para las importadas, tienen un impacto menor en el precio minorista final que si se aplican en función de una base imponible establecida en tramos posteriores de dicha cadena, como el precio minorista. Por otra parte, los impuestos selectivos de monto específico se aplican sobre la misma base imponible para las bebidas de producción local e importadas. Además, también reducen los incentivos para pasar a consumir marcas menos caras. No obstante, el valor real de los impuestos selectivos de monto específico y su eficacia para reducir el consumo tienden a disminuir con el tiempo si no son ajustados regularmente para tener en cuenta la inflación y también, idealmente, el aumento del ingreso (18).

Según la OMS, los impuestos selectivos calculados sobre la base del contenido de azúcar pueden tener una repercusión mayor. De hecho, crean una diferenciación en la carga impositiva entre las opciones basadas en el contenido de azúcar dentro de una categoría de productos y pueden inducir al consumidor a optar por bebidas de menor contenido de azúcar y, al mismo tiempo, alentar a los productores a reformular sus bebidas (5).

Partiendo del mismo razonamiento, desde el punto de vista de la salud, al agua embotellada no se le deberían aplicar impuestos selectivos, pues ello les resta a dichos impuestos la posibilidad de generar una diferencia de precio entre las bebidas azucaradas y las no azucaradas, y no induce al consumidor a abandonar las azucaradas en favor de una opción más saludable. Además, demuestra que, en algunos países, los impuestos selectivos sobre las bebidas azucaradas se han establecido para aumentar los ingresos fiscales, sin considerarlos como instrumento de políticas de salud.

Por último, la lista de productos imponibles debería incorporar todos los tipos de bebidas azucaradas, incluidas las bebidas lácteas azucaradas, así como los polvos, concentrados o jarabes usados para hacer bebidas azucaradas añadiendo agua no gasificada o gasificada, para prevenir las sustituciones no deseadas de bebidas azucaradas gravadas por otras no gravadas. La simplicidad y la transparencia en la estructura tributaria y la definición de los productos imponibles reducen las oportunidades de elusión fiscal.

### Oportunidades para centrarse en la salud

Habida cuenta de las consideraciones anteriores, los impuestos selectivos existentes se podrían modificar a fin de mejorar su eficacia para reducir el consumo de bebidas azucaradas, con medidas como recurrir más a los impuestos de monto específico, gravar en función del contenido de azúcar, aumentar las tasas tributarias, excluir el agua embotellada de la lista de productos imponibles y cerrar las lagunas que incentivan las sustituciones no deseables al incluir explícitamente en dicha lista todas las categorías de bebidas azucaradas: bebidas gaseosas azucaradas, bebidas con sabor a fruta, jugos de fruta, bebidas deportivas y energizantes, bebidas de agua vitaminada, tés helados y limonadas edulcoradas, bebidas lácteas y yogures azucarados, así como polvos, concentrados o jarabes usados para hacer bebidas azucaradas añadiendo agua sin o con gas.

En los países que no aplican impuestos selectivos sobre las bebidas azucaradas, la comunidad de salud pública tiene la oportunidad de abogar por su introducción y de asegurarse de que se diseñen de modo de optimizar los resultados de salud desde el principio.

### Necesidad de mejores prácticas en materia de tributación de las bebidas azucaradas

Entre los actuales impuestos selectivos sobre las bebidas azucaradas hay una gran heterogeneidad y poca orientación en cuanto a las mejores prácticas en la tributación de dichas bebidas en comparación con la orientación para la tributación del tabaco y las bebidas alcohólicas (18, 24). Claramente, hay que seguir elaborando mejores prácticas basadas en datos empíricos para diseños eficaces de impuestos selectivos sobre las bebidas azucaradas. Las investigaciones futuras deberían estar dirigidas a evaluar la repercusión que diferentes diseños de impuestos selectivos tienen sobre los precios, el consumo, los ingresos tributarios y los resultados de salud, y la medida en que distintos diseños se podrían vincular con consecuencias no deseadas.

### Necesidad de un seguimiento sistemático de la tributación de las bebidas azucaradas

Es necesario establecer sistemas de seguimiento periódico y estandarizado para captar los cambios en los niveles de tributación aplicados a las bebidas azucaradas en el transcurso del tiempo y poder efectuar comparaciones entre países. Para ello, partiendo de la experiencia de la OMS en cuanto al seguimiento de la tributación del tabaco, la OPS está trabajando en este momento a la elaboración de un indicador estandarizado sobre el porcentaje que los impuestos indirectos (incluidos los impuestos al valor agregado, selectivos y derechos aduaneros y de importación) representan en los precios minoristas de las bebidas azucaradas en América Latina y el Caribe (25).

### Limitaciones

En nuestro análisis no proporcionamos información sobre las tasas de los impuestos selectivos establecidas en la legislación. De hecho, las comparaciones entre países pueden resultar engañosas a menos que se tengan en cuenta las diferencias en cuanto a la estructura del impuesto, la base imponible, el precio, el contenido de azúcar y el volumen de la bebida. Si bien no nos centramos en las tasas establecidas de los impuestos selectivos, cabe destacar que dichas tasas deben ser suficientemente elevadas para ser un desincentivo efectivo que induzca al consumidor a no comprar bebidas azucaradas (5, 20, 26).

Aun cuando analizamos si los impuestos selectivos existentes se aplican especialmente a ciertas categorías de bebidas no alcohólicas, no hemos evaluado la tributación de las bebidas con sabor a fruta, los jugos de frutas o las bebidas deportivas. Ni dichos jugos ni las bebidas deportivas formaron parte de las bebidas para las cuales la encuesta de la OPS sobre los impuestos a las bebidas azucaradas recopiló información tributaria. Por otra parte, si bien dicha encuesta recogió información sobre la tributación de las bebidas con sabor a fruta, la información fue difícil de verificar en la legislación debido a la definición amplia del código arancelario armonizado, partida 2009, que incluye los jugos de fruta “con o sin adición de azúcar u otro edulcorante” (23).

Por último, los datos presentados en nuestro análisis están basados en legislación que estaba en vigor al 31 de marzo del 2019. La legislación que pudiera haber sido reemplazada, enmendada o derogada desde esa fecha de corte no fue analizada, de manera de mantener la comparabilidad de los datos en el mismo momento en todos los países. Desde esa fecha de corte no detectamos la introducción de ningún nuevo impuesto selectivo sobre las bebidas azucaradas en América Latina y el Caribe.

### Conclusión

Al mes de marzo del 2019, 21 países de América Latina y el Caribe aplicaban impuestos selectivos sobre las bebidas azucaradas. Si bien ello es prometedor, la mayoría de esos impuestos se podrían aprovechar aún más a fin de que tengan un mayor impacto sobre el consumo de bebidas azucaradas y la salud. Esto ofrece la oportunidad de proponer enmiendas a los impuestos selectivos existentes y abogar por su introducción en los países que actualmente no los aplican.

El diseño de los impuestos selectivos existentes sobre las bebidas azucaradas es sumamente diverso. Todos los países se beneficiarían de orientación adicional en cuanto a la manera de introducir o enmendar dichos impuestos para optimizar los beneficios de salud. Vista la carga mundial de las ENT tanto actual como proyectada, será importante dar estrecho seguimiento al uso de esa política eficaz a nivel poblacional y aumentar al máximo su potencial de salud.

## Declaración.

Los autores son los únicos responsables de las opiniones expresadas en el manuscrito, que pueden no reflejar necesariamente la opinión o la política de la *RPSP/PAJPH* o de la OPS.
